# Glyoxal in hyperglycaemic ischemic stroke – a cohort study

**DOI:** 10.1186/s12933-023-01892-7

**Published:** 2023-07-12

**Authors:** Sina Rhein, Julica Inderhees, Oliver Herrmann, Alaa Othman, Kimberly Begemann, Thomas Fleming, Peter P. Nawroth, Karel D. Klika, Rakad Isa, Inke R. König, Georg Royl, Markus Schwaninger

**Affiliations:** 1 Institute for Experimental and Clinical Pharmacology and Toxicology, Center for Brain, Behavior and Metabolism, University of Lübeck, Lübeck, Germany; 2 German Centre for Cardiovascular Research, (DZHK), Hamburg-Lübeck-Kiel, Germany; 3 Bioanalytic Core Facility, Center for Brain, Behavior and Metabolism, University of Lübeck, Lübeck, Germany; 4 Department of Neurology, University of Heidelberg, Heidelberg, Germany; 5 Department of Internal Medicine, University of Heidelberg, Heidelberg, Germany; 6 German Research Centre for Diabetes Research, Düsseldorf, Germany; 7 Molecular Structure Analysis, German Cancer Research Center (DKFZ), Heidelberg, Germany; 8 Department of Neurology, Center for Brain, Behavior and Metabolism, University of Lübeck, Lübeck, Germany; 9 Institute of Medical Biometry and Statistics, University of Lübeck, Lübeck, Germany

**Keywords:** Glucose, Advanced glycation end-products, Ischemic brain damage, Undernutrition

## Abstract

**Background:**

Hyperglycaemia is frequent in acute ischemic stroke and denotes a bad prognosis, even in the absence of pre-existing diabetes. However, in clinical trials treatment of elevated glucose levels with insulin did not improve stroke outcome, suggesting that collateral effects rather than hyperglycaemia itself aggravate ischemic brain damage. As reactive glucose metabolites, glyoxal and methylglyoxal are candidates for mediating the deleterious effects of hyperglycaemia in acute stroke.

**Methods:**

In 135 patients with acute stroke, we used liquid chromatography coupled to tandem mass spectrometry (LC-MS/MS) to measure glyoxal, methylglyoxal and several of their glycated amino acid derivatives in serum. Results were verified in a second cohort of 61 stroke patients. The association of serum concentrations with standard stroke outcome scales (NIHSS, mRS) was tested.

**Results:**

Glucose, glyoxal, methylglyoxal, and the glyoxal-derived glycated amino acid N_δ_-(5-hydro-4-imidazolon-2-yl)ornithine (G-H1) were positively correlated with a bad stroke outcome at 3 months as measured by mRS90, at least in one of the two cohorts. However, the glycated amino acids N_ε_-carboxyethyllysine (CEL) and in one cohort pyrraline showed an inverse correlation with stroke outcome probably reflecting lower food intake in severe stroke. Patients with a poor outcome had higher serum concentrations of glyoxal and methylglyoxal.

**Conclusions:**

The glucose-derived α-dicarbonyl glyoxal and glycated amino acids arising from a reaction with glyoxal are associated with a poor outcome in ischemic stroke. Thus, lowering α-dicarbonyls or counteracting their action could be a therapeutic strategy for hyperglycaemic stroke.

**Supplementary Information:**

The online version contains supplementary material available at 10.1186/s12933-023-01892-7.

## Background

**Fig. 1:**
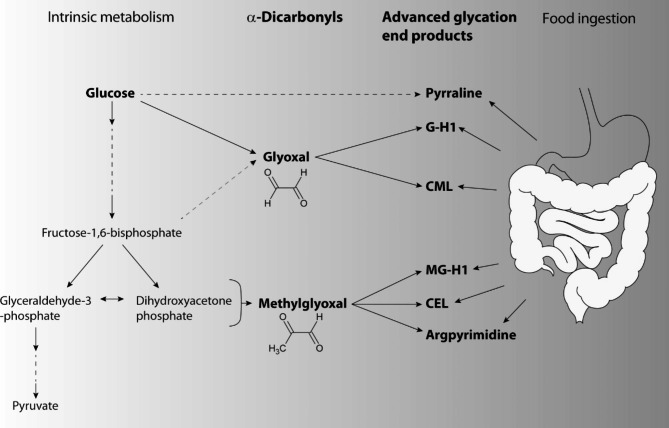
Schematic illustration of the connection of glucose metabolism, α-dicarbonyl production, and formation of related glycated amino acids [60]. While glyoxal and pyrraline can be generated directly from glucose by autooxidation, glycolysis is necessary to provide the methylglyoxal precursor molecules glyceraldehyde-3-phosphate and dihydroxyacetone phosphate. α-Dicarbonyls modify arginine or lysine residues resulting in the formation of G-H1 and CML or MG-H1, CEL and argpyrimidine through the reaction with glyoxal and methylglyoxal, respectively. Furthermore, glycated amino acids can be absorbed after digestion of food

Research has focused on the harmful effects of hyperglycaemia on some organs, including kidney, retina or heart. Less is known about the impact of hyperglycaemia on the brain, despite its frequent association with stroke. Being a major risk factor for stroke, diabetes mellitus partially explains why hyperglycaemia is diagnosed in up to 75% of patients with acute stroke [1]. However, even without pre-existing diabetes mellitus, hyperglycaemia is frequent in stroke patients during the acute phase. In ischemic stroke, hyperglycaemia on admission is associated with a poor neurological outcome, an increased risk of symptomatic intracranial haemorrhage, and enhanced mortality [2–4]. Importantly, several studies have shown that in nondiabetic stroke patients hyperglycaemia revealed an even stronger predictive value [5–7]. Together with preclinical data, this provides evidence that acute hyperglycaemia is detrimental for functional recovery, but the mechanisms and molecular mediators by which acute hyperglycaemia influences the neurological outcome are still unclear [8]. Candidates to worsen the outcome in hyperglycaemia are α-dicarbonyls, a group of glucose metabolites, prominent representatives being glyoxal and methylglyoxal (Fig. 1). Glyoxal is mainly generated via glucose autoxidation while methylglyoxal is a by-product of glycolysis [9, 10]. Another source for α-dicarbonyls is the glycation of proteins. Condensation reactions of free amino groups of proteins with reducing sugars, like glucose, lead to the formation of Schiff bases and subsequent Amadori rearrangements. Both Schiff bases and Amadori products undergo further rearrangements, resulting in α-dicarbonyls, like glyoxal and methylglyoxal [11]. Glyoxal and methylglyoxal share a highly reactive chemical structure and are able to modify proteins, DNA and lipids, thereby impeding the function of various macromolecules [12]. The products of their reaction with protein residues like lysine and arginine are referred to as advanced glycation end products (AGEs), which may also be derived from Amadori products [13, 14].

Modification of proteins by α-dicarbonyls and the interaction of AGEs with the receptor for AGEs (RAGE) increase oxidative stress and create a proinflammatory environment [15, 16]. In rodents, several studies have revealed detrimental effects of α-dicarbonyls, AGEs, or RAGE signalling on the outcome after experimental stroke [17–22].

In human hyperglycaemic stroke, however, evidence for the involvement of α-dicarbonyls and AGEs is less clear. Glycation of proteins in human samples can be investigated by tryptic hydrolysis of proteins and enrichment of glycated peptides, followed by mass spectrometry [23–26]. Another way of monitoring AGE production is to measure blood concentrations of glycated amino acids that are endogenously formed by proteolysis of AGEs or by the reaction of α-dicarbonyls with free amino acids (Fig. 1) [27]. However, glycated amino acids are also ingested with the diet [28–31]. As patients often remain fasting during the acute phase of stroke, it is unclear whether specific glycated amino acids rise due to hyperglycaemia or fall due to fasting. Several methods are available for the detection of α-dicarbonyls and glycated amino acids. Immunoassays and the measurement of skin autofluorescence, which reflects the presence of glycation adducts, are subject to numerous technical errors [27]. The best sensitivity and the highest specificity is achieved by liquid chromatography coupled to tandem mass spectrometry (LC-MS/MS) [27]. So far, the few clinical studies that have investigated AGEs or individual glycated amino acids in acute stroke patients have used immunoassays or the measurement of skin autofluorescence [32–34]. Thus, concentrations of α-dicarbonyls or glycated amino acids and their association with neurological outcome after hyperglycaemic stroke is still unclear.

Therefore, we performed a quantitative analysis of the α-dicarbonyls glyoxal and methylglyoxal and the corresponding glycated amino acids N_δ_-(5-hydro-4-imidazolon-2-yl)ornithine (G-H1), N_δ_-(5-hydro-5-methyl-4-imidazolon-2-yl)ornithine (MG-H1), N_ε_-carboxymethyllysine (CML), N_ε_-carboxyethyllysine (CEL), argpyrimidine, and pyrraline in serum samples of acute stroke patients. The primary objective was to determine whether these reactive glucose metabolites accumulate in stroke patients with an adverse outcome, indicating that they may cause the known toxic effects associated with hyperglycaemia.

## Methods

### Patients/Subjects/Cohorts

Acute ischemic stroke within the prior 4 days was the inclusion criterion. We collected blood samples from patients on admission (cohort 1 and 2) and on the three consecutive days until discharge (cohort 1). Due to early discharge or other reasons, some patients were lost to follow-up (Additional File 1 Table S1, Additional File 1 Table S2). The time between the last meal and blood sampling was documented according to the information provided by the patients or accompanying persons. The diagnosis of diabetes or other risk factors was based on the history.

### Detection of α-dicarbonyls, glycated amino acids and glucose

Samples were stored at -80° C before analysis. Samples from cohort 1 and cohort 2 were measured in separate experiments by the same laboratory. α-Dicarbonyls were extracted as described previously with some modifications [20]. For protein precipitation, 40 µl of ice-cold trichloroacetic acid (20%, w/v, Sigma-Aldrich) was added to 100 µl of serum. After adding 80 µl water and 20 µl of the isotopically labelled internal standard deuterated methylglyoxal (d4-MG, 400 nM in H_2_O), the sample was incubated on ice for 10 min followed by centrifugation (20,000 x g, 4° C, 10 min). The supernatant was transferred to a glass vial, and the α-dicarbonyls were derivatized to the respective quinoxaline compounds with isotopically labelled d8-o-phenylenediamine (CDN Isotopes, 10 µl, 0.5 mM in 200 mM HCl/500 µM diethylenetriaminepentaacetic acid (DETAPAC, Sigma-Aldrich) for 4 h at room temperature in the dark. D4-MG was synthesized, as previously described [35], using d6-acetone. The concentration and purity of the d4-MG stock solutions, as well as the identities of the contaminants, were determined from 1 H, 2 H and 13 C NMR spectra acquired at 298 K using a Bruker Avance II NMR spectrometer. The purities of the d4-MG stock solutions were 60–65% based on the integration of the signals in the 2 H NMR spectrum, adjusted according to the number of equivalent nuclei with the major contaminants being d3-acetate and d6-acetone. Representative spectra are shown in Additional File 1 Fig. S1.

Since α-dicarbonyls are highly reactive, we tested in a pilot study whether small differences in the time between blood collection and sample extraction or in the protocol of blood extraction could influence sample concentrations. Systematic variation of these parameters did not significantly affect results (Additional File 1 Fig. S2) suggesting that our protocol for α-dicarbonyl measurement is robust.

For the detection of glycated amino acids, 360 µl of acetonitrile with 0.1% formic acid (Biosolve) was added to 100 µl serum for protein precipitation. After the addition of 10 µl internal standard mix (d2-CML, d4-CEL, d3-MG-H1, 400 nM in H_2_O, PolyPeptide Group), the samples were mixed thoroughly and incubated for 30 min at -20 °C. After centrifugation (20,000 x g, 4° C, 10 min), the supernatant was transferred to glass vials for detection.

LC/MS analysis was performed on a TSQ Endura triple quadrupole mass spectrometer, equipped with a heated electrospray ionization source and coupled to a Dionex Ultimate 3000 UHPLC system (ThermoFisher Scientific, Bremen, Germany). For both methods water containing 0.1% formic acid as mobile phase A and 100% acetonitrile as phase B were used. All solvents were of LC-MS grade quality and were purchased from Merck (Darmstadt, Germany).

α-Dicarbonyls were measured as described previously with some modifications [20]. Briefly, quinoxaline derivatives were separated on an Ascentis Express C18 column (100 mm x 2.1 mm x 5 µM; Sigma-Aldrich) applying an isocratic gradient of 90% A and 10% B with a flow rate of 0.2 ml/min for 10 min. Afterwards, B increased to 100% between 10 and 11 min, which was followed by a washing step with 100% B between 11 and 15 min with a flow rate of 0.4 ml/min. Re-equilibration took place between 15 and 20 min back to 10% B. Following parameters were used in the positive ionization mode: ion spray voltage, 4600 V; vaporizer temperature, 100 °C; and ion transfer tube temperature, 300 °C.

For glycated amino acids, a BEH amide column was used (XBridge BEH Amide, 100 mm x 2.1 mm, 2.5 μm, Waters). The chromatographic separation was realized using following gradient at 40 °C and a flow rate of 0.4 ml/min: 95% B from 0 to 3 min, followed by a decrease to 60% B from 3 to 6 min and to 30% B from 6 to 12 min. After washing at 30% B from 12 to 16 min, re-equilibration took place by increasing to 95% B from 16 to 25 min and keeping 95% for another 5 min. Following parameters were used in the positive ionization mode: ion spray voltage, 4000 V; vaporizer temperature, 200 °C; and ion transfer tube temperature, 300 °C.

Multiple reaction monitoring (MRM) was used to identify quinoxaline derivates and glycated amino acids with collision-induced fragmentation at 2.5 mTorr using argon. Retention times, MRM transitions, scan parameters, limits of quantification, and inter batch variances are listed in Additional File 1 Table S3. Representative spectra are shown in Additional File 1 Fig. S3. Quantification was performed with an external calibration curve based on the ratio of the areas under the peaks to the internal standard. Each sample was measured as a singleton. Every 20–30 samples, one quality control sample was included in the measurements.

Glucose concentrations were measured in venous serum samples using the Glucose-Glo™ Assay (Promega, USA) according to the manufacturer’s instruction.

### Statistics

To identify potential associations of glyoxal and methylglyoxal as well as glycated amino acids with stroke outcome and hyperglycaemia, we chose an explorative approach. For correlation analysis we used the Spearman test. Group differences were analysed using the Mann Whitney U-test. Stroke patients were stratified according to mRS90 values into good (0–2) and bad (3–6) outcome groups. Odds ratios (OR) for a bad outcome were estimated by defining groups according to the median concentration of glucose, α-dicarbonyls, or glycated amino acids (< or > = median). Categorical variables were compared with the Chi-square test.

To investigate whether glyoxal at day 2 was associated with outcome, we predicted outcome based on the logistic regression model by Weimar et al. that uses age and NIHSS at admission [36]. Overall accuracy was 82.9%, sensitivity 75%, and specificity 84.8%. We included Box-Cox transformed glyoxal concentrations as an additional covariable and compared both model fits using a likelihood-ratio test. Using fractional polynomials, we found no indication that the relationship between transformed glyoxal and outcome is non-linear. Finally, we identified no interactions between glyoxal and age as well as NIHSS in predicting outcome.

## Results

**Fig. 2:**
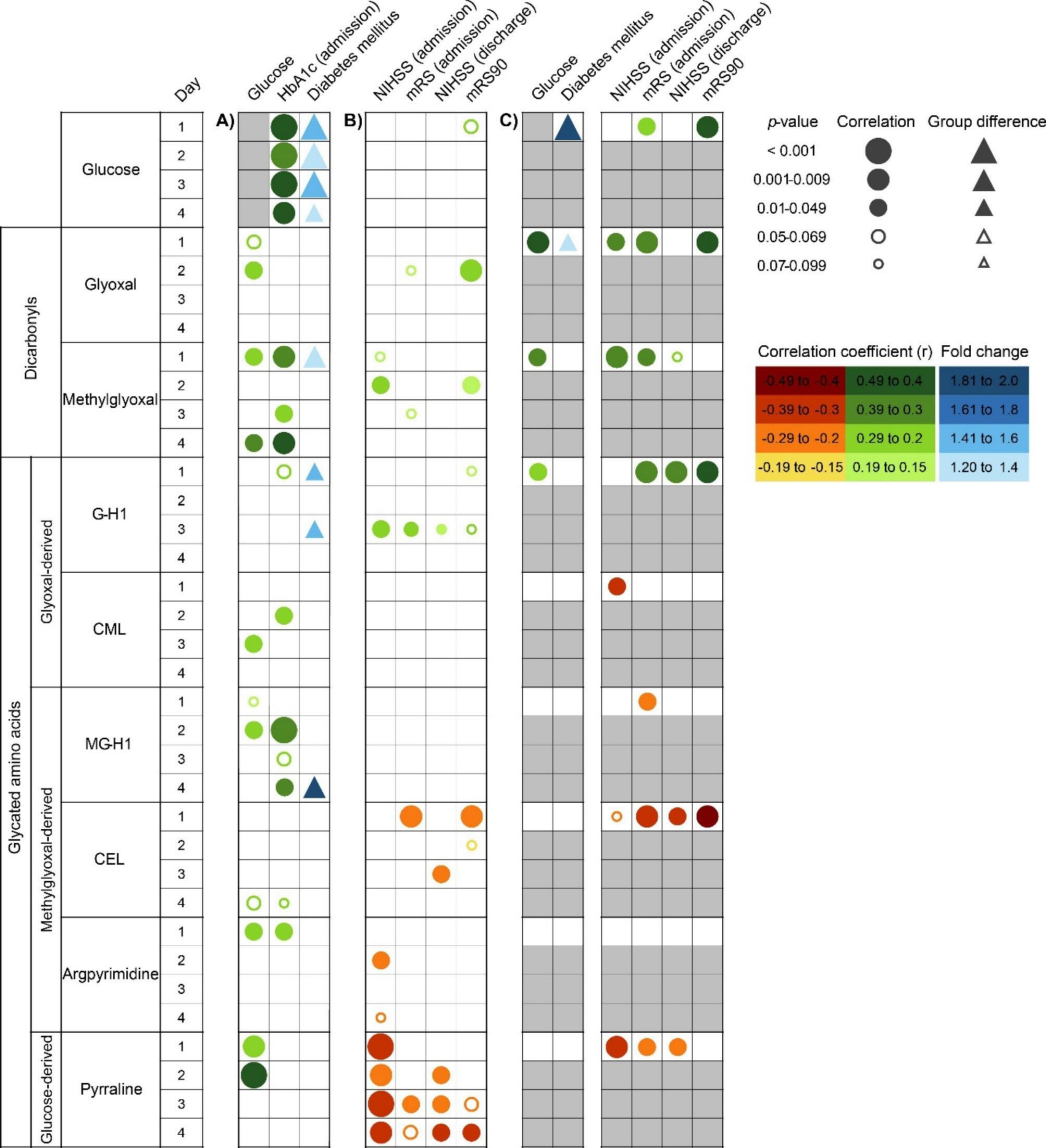
P-value heatmap of correlations and group differences of glucose, α-dicarbonyls, and glycated amino acids with diabetes-related parameters and neurological scores. Blood samples of four consecutive days after stroke have been analysed using LC-MS. (A) In patients of cohort 1, concentrations of the compounds were tested for an association with the history of diabetes or laboratory parameters of hyperglycaemia and diabetes (glucose, HbA1c, Spearman coefficient; diabetes mellitus, Mann Whitney U-test). (B) Correlations with neurological scores on admission (mRS, NIHSS) and later stages (mRS day 90, NIHSS discharge) in cohort 1 (Spearman coefficient). (C) Results were confirmed in cohort 2. mRS, modified Rankin Scale; NIHSS, National Institutes of Health Stroke Scale. White squares indicate no correlation, grey squares indicate parameters that were not measured (day 2–4, cohort 2) or for which a correlation with themselves (glucose) was meaningless

For the investigation of α-dicarbonyls and their derivatives, we recruited 135 patients with acute ischemic stroke in cohort 1. Demographic data and clinical characteristics are presented in Additional File 1 Table S1. The median time between stroke onset and first blood sampling was 13.2 h (IQR = 6.3 − 20.0 h) (day 1). In 51 of 102 patients, for whom measurements in venous blood samples were available on admission, glucose concentrations were above 7.8 mM (140 mg/dl), including 20 diabetes mellitus patients and 31 patients without diagnosed diabetes (Additional File 1 Fig. S4). We analysed concentrations of α-dicarbonyls and glycated amino acids in relation to neurological scores or related metabolic parameters; the results are presented as a p-value heatmap (Fig. 2). As expected, α-dicarbonyls and glycated amino acids positively correlated with concentrations of glucose or HbA1c and were elevated in subjects with diabetes (Fig. 2A).

Interestingly, glyoxal, methylglyoxal and the glyoxal-derived G-H1 positively correlated with mRS or NIHSS on admission or the NIHSS at discharge from stroke unit (median, 4 days after onset of symptoms, Fig. 2B). In contrast, the methylglyoxal-derived glycated amino acids CEL, argpyrimidine and pyrraline showed a negative correlation, if any, with the mRS or NIHSS on admission or discharge from stroke unit (Fig. 2B). Food contains high amounts of AGEs which, after ingestion and digestion, reach the blood stream as glycated amino acids [28–31], but many patients do not eat because of dysphagia after stroke. In support of a dietary source, pyrraline and MG-H1 concentrations on day 1 inversely correlated with the time since the last meal of patients (Additional File 1 Fig. S5A). Furthermore, on day 3, pyrraline showed a positive correlation with 4-hydroxyproline, a marker for the intake of meat, which is a rich source of AGEs (Additional File 1 Fig. S5B) [37–39]. The inverse correlation between CEL, argpyrimidine and pyrraline and neurological scores at early timepoints could therefore be due to impaired oral food intake of patients with a severe stroke [40–42].

In line with the previously reported association between post-stroke hyperglycaemia and bad outcome, glucose concentrations on day 1 showed a trend toward correlation with mRS90 (p = 0.052, Fig. 2B) [2, 8, 43–45]. Interestingly, we also observed a positive correlation of glyoxal and methylglyoxal concentrations on day 2 with mRS90 (Fig. 2B). The delay may be accounted for by the time needed for the production of α-dicarbonyls from glucose. In addition, the glyoxal-derived glycated amino acid G-H1 on day 3 of admission showed a trend to a correlation with mRS90 values. In contrast, the methylglyoxal-derived glycated amino acids MG-H1, CEL and argpyrimidine were not correlated with mRS90 or were negatively correlated with mRS90 values (Fig. 2B). Pyrraline levels on day 3 and 4 were also inversely correlated with mRS90. In patients with a favourable outcome after stroke, high pyrraline concentrations may reflect early normalization of food intake.

We re-evaluated the association between α-dicarbonyls or glycated amino acids and stroke outcome in cohort 2 which included 61 acute ischemic stroke patients (demographic characteristics in Additional File 1 Table S2). Similar to the results of cohort 1, glyoxal, methylglyoxal, and G-H1 positively correlated with NIHSS or mRS on day 1, whereas MG-H1, CEL, and pyrraline showed a negative correlation (Fig. 2C). Again, glyoxal and G-H1 concentrations positively correlated with mRS90.

To assess the prognostic value of the various parameters, we dichotomized patients of cohort 1 based on serum concentrations either above or below the median (Additional File 1 Fig. S6). With a glucose concentration on day 1 above the median of 7.8 mmol/l, patients had an OR of 3.28 (95% CI 1.14–9.46, p = 0.023) for a poor outcome. Among the α-dicarbonyls, only high glyoxal concentrations on day 2 denoted a higher OR for a bad outcome, while there was no effect for methylglyoxal. Interestingly, increased levels of glyoxal-related glycated amino acids G-H1 and CML on day 1 and 2, respectively, also increased the risk for a bad outcome (Additional File 1 Fig. S6). Similarly, in cohort 2 high levels of G-H1 elevated the odds ratio for a poor outcome (Additional File 1 Fig. S7). In contrast, high concentrations of the methylglyoxal-related CEL on day 4 or pyrraline on day 3 were associated with a lower risk for a bad outcome, at least in cohort 1 (Additional File 1 Fig. S6). Overall, glyoxal and related glycated amino acids had the strongest association with a bad outcome after ischemic stroke. However, in a clinical prognostic model based on age and NIHSS [36], adding glyoxal concentrations on day 2 did not improve the prediction of outcome. This may be due to the fact that glyoxal plasma concentrations correlated with age.

**Fig. 3:**
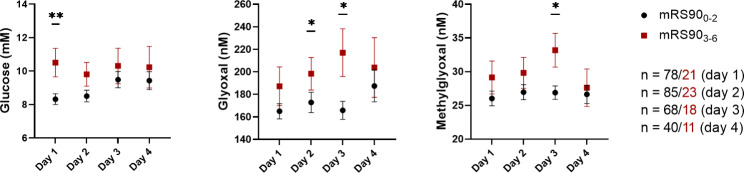
Timeline of serum levels of patients in cohort 1 with a good or bad outcome. Concentrations of glucose, glyoxal and methylglyoxal were compared between good (mRS90_0 − 2_) and bad outcome groups (mRS90_3 − 6_). Mean ± SEM. *, p < 0.05, **, p < 0.01 (Mann Whitney U test)

When comparing patients from cohort 1 with a poor outcome (mRS90 ≥ 3) and a good outcome (mRS90 ≤ 2), we found higher serum concentrations of glucose on day 1, glyoxal and methylglyoxal on day 2 or 3 in the poor outcome group (Fig. 3, Additional File 1 Fig. S8). The sequence in which changes occurred may reflect the conversion of glucose to α-dicarbonyls. Because patients with a poor outcome were more likely to have a history of diabetes, the analysis was repeated after excluding diabetes patients. Despite the lower numbers of cases, most of the differences between the prognostic groups remained in patients without known diabetes, arguing that the metabolites reflect the acute stroke-induced hyperglycaemia (Additional File 1 Fig. S9). The analysis of cohort 2 confirmed elevated serum concentrations of glucose, glyoxal, and methylglyoxal in patients with a poor outcome (Additional File 1 Fig. S10).

## Discussion

Hyperglycaemia after stroke is associated with a poor outcome and experimental evidence indicates that high blood levels of glucose aggravate ischemic brain damage [2–4, 8]. To explore the possibility that glucose worsens stroke outcome through reactive α-dicarbonyl metabolites and the resulting α-dicarbonyl stress, we measured the reactive glucose metabolites glyoxal and methylglyoxal as well as their glycated amino acid derivatives in acute stroke patients. For the analysis, we used a highly sensitive and specific LC-MS/MS approach. Prior to detection dicarbonyls were derivatised to increase the sensitivity, as previously described [46–48]. In line with earlier reports [2, 5–7], glucose concentrations on admission correlated with poor functional outcome after 3 months as measured by mRS90. Similarly, we found a correlation for glyoxal, methylglyoxal and the glyoxal-derived glycated amino acid G-H1 with mRS90 when investigating two independent cohorts of acute stroke patients. The association with a bad outcome as defined by mRS90 > 2 was strongest for glyoxal. However, the relationship between methylglyoxal and a bad outcome was not very robust. Differences between glyoxal and methylglyoxal are likely due to the way of how they are produced from glucose. While glyoxal is formed extracellularly by autoxidation from glucose, methylglyoxal is a by-product of intracellular glycolysis (Additional File 1 Fig. S11). After stroke, glucocorticoids and inflammatory mediators induce insulin resistance and hyperglycaemia [2]. We propose that insulin resistance and the blockage of glucose entry into insulin-sensitive tissues favour extracellular glyoxal production in plasma from glucose which consequently leads to the formation of glyoxal-associated glycated amino acids [2, 49].

α-Dicarbonyls are involved in the pathogenesis of atherosclerotic disease and are associated with a higher risk of stroke [50, 51]. In acute stroke, hyperglycaemia and other factors, such as hypoxia or lipid peroxidation, may increase the production of α-dicarbonyls and AGEs. In preclinical models, glyoxal and glyoxal-derived AGEs aggravate ischemic brain damage by RAGE-dependent or -independent mechanisms [19, 20, 52]. The AGEs receptor RAGE is expressed by monocyte-derived macrophages and determines their polarization towards an inflammatory cell type [19, 20]. In addition, activation of RAGE mediates the metabolic reprogramming of T cells and skews their differentiation in experimental stroke models [52]. These mechanisms may explain that RAGE mediates the deleterious effects of hyperglycaemia in stroke [20]. In addition, dicarbonyls have RAGE-independent effects on brain cells. In brain endothelial cells, they downregulate the expression of CSF-1 that induces a noninflammatory polarization of macrophages in the ischemic brain [20]. Glyoxal exposure has been reported to lead to the collapse of the mitochondrial membrane potential and compromise the survival of endothelial cells [53]. Toxic effects have also been found in neuronal cells with glyoxal and glyoxal-derived AGEs inducing apoptosis [54–56]. The correlation of glyoxal concentrations with a poor prognosis that we have observed in this study suggests that the proinflammatory and neurotoxic effects of glyoxal and glyoxal-derived AGEs are relevant in acute hyperglycaemic stroke.

Unexpectedly, although methylglyoxal showed a weak positive correlation with poor stroke outcome, methylglyoxal-derived glycated amino acids and pyrraline in serum inversely correlated with poor stroke outcome. This finding is likely due to the reduced food intake in patients with a severe stroke [40–42]. Food is a rich source of AGEs that are degraded to glycated amino acids and absorbed into the blood stream [28–31, 57]. In support of this explanation, pyrraline and MG-H1 inversely correlated with the time since the last meal. Whether α-dicarbonyls or glycated amino acids derived from AGEs in food have a detrimental effect on health, is still a contentious matter [57]. The fact that undernutrition in stroke is known to be associated with a poor outcome suggests that food-derived α-dicarbonyls or glycated amino acids do not have a strong effect on ischemic brain injury [40, 41].

The present study has some limitations. One problem relates to patient attrition. As not all patients remained on the stroke unit until day 4, the time course of concentrations has to be interpreted with caution. Furthermore, the overall number of patients was rather small, so we may have overlooked confounding factors. However, importantly, we found similar results in two independent cohorts of stroke patients. Obviously, the association between glyoxal and outcome does not prove causality. Interventions studies will be required to address this point.

## Conclusions

As lowering elevated glucose concentrations with insulin had no significant effect on stroke prognosis [58], other strategies are needed to prevent the neurotoxic effects of glucose. Indeed, the formation of α-dicarbonyls or their action provides a rich treasure trove for potential pharmacological targets [59]. In preclinical stroke models, several of these interventions reduced ischemic brain damage [17, 19, 52]. Our results suggest that efforts to translate this strategy into the clinic and reduce α-dicarbonyls or counteract their effects may be successful in the treatment of acute stroke.

## Electronic supplementary material

Below is the link to the electronic supplementary material.

Supplementary Material 1

## Data Availability

The datasets analysed during the current study are available from the corresponding author on reasonable request.

## Abbreviations

CEL: Nε-carboxyethyllysine
CML: Nε-carboxymethyllysine
G-H1: Nδ-(5-hydro-4-imidazolon-2-yl) ornithine
LC-MS/MS: Liquid chromatography coupled to tandem mass spectrometry
MG-H1: Nδ-(5-hydro-5-methyl-4-imidazolon-2-yl) ornithine
MRM: Multiple reaction monitoring
NIHSS: National Institute of Health stroke scale
mRS: Modified Rankin scale
RAGE: Receptor for AGEs
