# Supplementary material for: Glyoxal in hyperglycaemic ischemic stroke – a cohort study
Source: Cardiovasc Diabetol. 2023 Jul 12;22:173. doi: 10.1186/s12933-023-01892-7 (PMC10339542; doi:10.1186/s12933-023-01892-7)
Supplement: Supplementary file 1 — Supplementary Material 1 [file 12933_2023_1892_MOESM1_ESM.docx]

**Additional File 1**

**Table S1** Baseline characteristics of stroke patients in cohort 1. Eight patients were unavailable to follow-up after 90 days. P values refer to the comparison of patients with mRS90_0-2_ and mRS90_3-6_.

| **Baseline characteristics [unit]** | **Ischemic stroke patients (n=135)** | **Ischemic stroke patients with follow-up at 90 days (n=127)** | | ***P* value** (mRS90_0-2_ vs mRS90_3-6_) |
| --- | --- | --- | --- | --- |
|  |  | **mRS90_0-2_ (n=101)** | **mRS90_3-6_ (n=26)** |  |
| Age [years] | 71.0  (61.0, 78.0) | 69.0  (57.5, 76.0) | 78.5  (74.5, 82.3) | **<0.001** |
| Women | 32/135 (23.7) | 20/101 (19.8) | 7/26 (26.9) | 0.429 |
| Body weight [kg] | 80.0  (70.0, 87.0) | 80.0  (72.5, 87.5) | 82.0  (69.5, 88.5) | 0.811 |
| BMI [kg/m^2^] | 26.4  (24.0, 28.6) | 26.8  (24.1, 29.2) | 26.8  (23.8, 28.4) | 0.618 |
| Abdominal/hip girth | 0.95  (0.93, 0.96) | 0.95  (0.93, 0.96) | 0.95  (0.93, 0.96) | 0.765 |
| History of diabetes | 31/132 (23.5) | 18/101 (17.8) | 11/26 (42.3) | **0.006** |
| … hypertension | 96/135 (71.1) | 68/101 (67.3) | 21/26 (80.8) | 0.182 |
| … AF | 24/134 (17.9) | 14/101 (13.9) | 7/26 (26.9) | 0.261 |
| … carotis OP/ stenting | 17/134 (12.7) | 11/101 (10.9) | 6/26 (23.1) | 0.108 |
| … stroke | 29/134 (21.6) | 16/101 (15.8) | 10/26 (38.5) | **0.012** |
| … myocardial infarction | 18/134 (13.4) | 10/101 (9.9) | 8/26 (30.8) | **0.007** |
| … CHD | 22/133 (16.5) | 15/101 (14.9) | 6/26 (23.1) | 0.325 |
| … PAD | 11/133 (8.3) | 8/101 (7.9) | 3/26 (11.5) | 0.569 |
| … smoking currently  …smoking previous | 19/135 (14.1)  45/135 (33.3) | 16/101 (15.8)  35/101 (34.7) | 0/26 (0.0)  8/26 (30.8) | **0.030**  0.709 |
| …alcohol consumption (medium to heavy) | 82/135 (60.7) | 62/101 (61.4) | 14/26 (53.8) | 0.484 |
| … hyper-cholesterolemia | 48/134 (35.8) | 32/101 (31.7) | 13/26 (50.0) | 0.095 |
| Time onset – first blood sampling [h] | Day 1 (D1): 13.2 (6.3, 20.0),  n = 104  Day 2 (D2): 37.0 (26.5, 43.9),  n = 112  Day 3 (D3): 62.2 (50.4, 68.0),  n = 91  Day 4 (D4): 88.3 (82.0, 94.8),  n = 55 | D1: 14.2  (6.9, 20.5),  n = 78  D2: 37.0  (26.8, 44.5),  n = 85  D3: 62.2  (51.0, 69.0),  n = 68  D4: 88.4  (82.0, 95.1),  n = 40 | D1: 11.0  (5.1, 19.0),  n = 21  D2: 36.0  (25.0, 42.5),  n = 23  D3: 61.4  (49.0, 67.2),  n = 18  D4: 86.5  (77.2, 91.0),  n = 11 | 0.351  0.403  0.625  0.233 |
| Time since last meal [h] | 13.6  (1.3, 16.2) | 10.6  (1.25, 15.5) | 14.4  (4.4, 18.9) | 0.174 |
| Time until discharge from hospital [days] | 4.0  (3.0, 6.5) | 4.0  (3.0, 6.8) | 5.0  (3.5, 7.0) | 0.447 |
| Thrombolysis treatment | 34/135 (25.2) | 26/101 (25.7) | 6/26 (23.1) | 0.780 |

Values are median (25%, 75% percentile), or n/total (%) as appropriate. BMI, body mass index; AF, atrial fibrillation; CHD, coronary heart disease, PAD, peripheral artery disease. For continuous data, groups were compared with the Mann Whitney U-test. For categorical variables, we used the Chi-square test.

**Table S2**: Baseline characteristics of stroke patients in cohort 2. Nineteen patients were unavailable to follow-up after 90 days. P values refer to the comparison of patients with mRS90_0-2_ and mRS90_3-6_.

| **Baseline characteristics**  **[unit]** | **Ischemic stroke patients**  **(n=61)** | **Ischemic stroke patients with follow-up at 90 days (n=42)** | | ***P* value** (mRS90_0-2_ vs mRS90_3-6_) |
| --- | --- | --- | --- | --- |
|  |  | **mRS90_0-2_ (n=17)** | **mRS90_3-6_ (n=25)** |  |
| Age [years] | 74.0  (61.5, 83.0) | 70.0  (59.5, 80.0) | 79.0  (70.0, 85.5) | **0.043** |
| Women | 28/61 (45.9) | 8/17 (47.1) | 16/25 (64.0) | 0.348 |
| History of diabetes | 6/61 (9.8) | 1/17 (5.9) | 4/25 (16.0) | 0.638 |
| … hypertension | 46/61 (75.4) | 8/17 (47.1) | 22/25 (88.0) | **0.006** |
| … AF | 22/61 (36.1) | 4/17 (23.5) | 9/25 (36.0) | 0.505 |
| … dementia | 5/61 (8.2) | 0/17 (0) | 3/25 (12.0) | 0.260 |
| … stroke | 19/61 (31.1) | 3/17 (17.6) | 8/25 (32.0) | 0.477 |
| … CHD | 13/61 (21.3) | 1/17 (5.9) | 7/25 (28.0) | 0.114 |
| … PAD | 5/61 (8.2) | 3/17 (17.6) | 1/25 (4.0) | 0.286 |
| … smoking currently | 11/41 (26.8) | 8/14 (57.1) | 0/13 (0.0) | **0.002** |
| …hyper-cholesterolemia | 33/60 (55.5) | 9/17 (52.9) | 15/25 (60.0) | 0.755 |
| Time until discharge from hospital [days] | 9.0  (7.0, 12.0) | 7.0  (7.0, 11.5) | 10.0  (8.0, 13.5) | **0.036** |
| Lysis | 11/61 (18.0) | 4/17 (23.5) | 5/25 (20.0) | 0.999 |
| Thrombectomy | 15/61 (24.6) | 5/17 (29.4) | 8/25 (32.0) | 0.999 |

Values are median (25%, 75% percentile), or n/total (%) as appropriate. BMI, body mass index; AF, atrial fibrillation; CHD, coronary heart disease, PAD, peripheral artery disease. For continuous data, groups were compared with the Mann Whitney U-test. For categorical variables, we used the Chi-square test.

**Table S3**: Technical data for the detection of α-dicarbonyls and glycated amino acids using mass spectrometry. Coefficient of variation (CV%), limit of detection (LOD) and limit of quantification (LOQ) were calculated based on the measurement of cohort 1.

| Substance | Internal Standard | Inter batch  CV% | LOQ  (pmol) | LOD (pmol) | Retention time (min) | Precursor ion (m/z) | Frag-ment ion (m/z) | Collision energy (eV) |
| --- | --- | --- | --- | --- | --- | --- | --- | --- |
| Glyoxal | d4-MG | 18.40 | 1.01 | 0.30 | 6.2 | 135.17 | 81.00 | 30.00 |
|  |  |  |  |  |  |  | 108.10 | 21.00 |
| Methyl-glyoxal | d4-MG | 6.01 | 0.12 | 0.04 | 7.2 | 149.10 | 81.27 | 31.00 |
|  |  |  |  |  |  |  | 122.16 | 23.00 |
| d4-Methyl-glyoxal | - | 6.08 | - | - | 7.2 | 153.17 | 81.27 | 32.00 |
|  |  |  |  |  |  |  | 125.10 | 24.00 |
| CML | d2-CML | 13.61 | 2.32 | 0.70 | 7.4 | 205.90 | 84.22 | 17.74 |
|  |  |  |  |  |  |  | 130.22 | 10.25 |
| CEL | d4-CEL | 12.84 | 1.23 | 0.37 | 7.3 | 219.10 | 84.22 | 19.30 |
|  |  |  |  |  |  |  | 130.11 | 10.25 |
| G-H1 | d3-  MG-H1 | 18.89 | 2.00 | 0.60 | 7.3 | 215.08 | 70.28 | 21.58 |
|  |  |  |  |  |  |  | 116.11 | 11.90 |
| MG-H1 | d3-  MG-H1 | 22.02 | 2.17 | 0.65 | 7.1 | 229.1 | 70.29 | 23.45 |
|  |  |  |  |  |  |  | 114.11 | 14.35 |
| Argpyri-midine | d3-  MG-H1 | 30.36 | 2.88 | 0.87 | 6.9 | 255.12 | 70.26 | 26.65 |
|  |  |  |  |  |  |  | 140.11 | 15.76 |
|  |  |  |  |  |  |  | 192.05 | 18.56 |
| Pyrraline | d4-CEL | 43.63 | 5.33 | 1.60 | 6.1 | 255.10 | 84.22 | 26.03 |
|  |  |  |  |  |  |  | 148.11 | 17.48 |
| d2-CML | - | 29.00 | - | - | 7.4 | 207.10 | 84.22 | 17.53 |
|  |  |  |  |  |  |  | 130.11 | 10.25 |
| d4-CEL | - | 26.27 | - | - | 7.3 | 223.13 | 88.22 | 19.25 |
|  |  |  |  |  |  |  | 134.10 | 10.25 |
| d3-MG-H1 | - | 18.02 | - | - | 7.1 | 232.10 | 70.26 | 22.79 |
|  |  |  |  |  |  |  | 117.10 | 13.15 |

**Fig. S1**


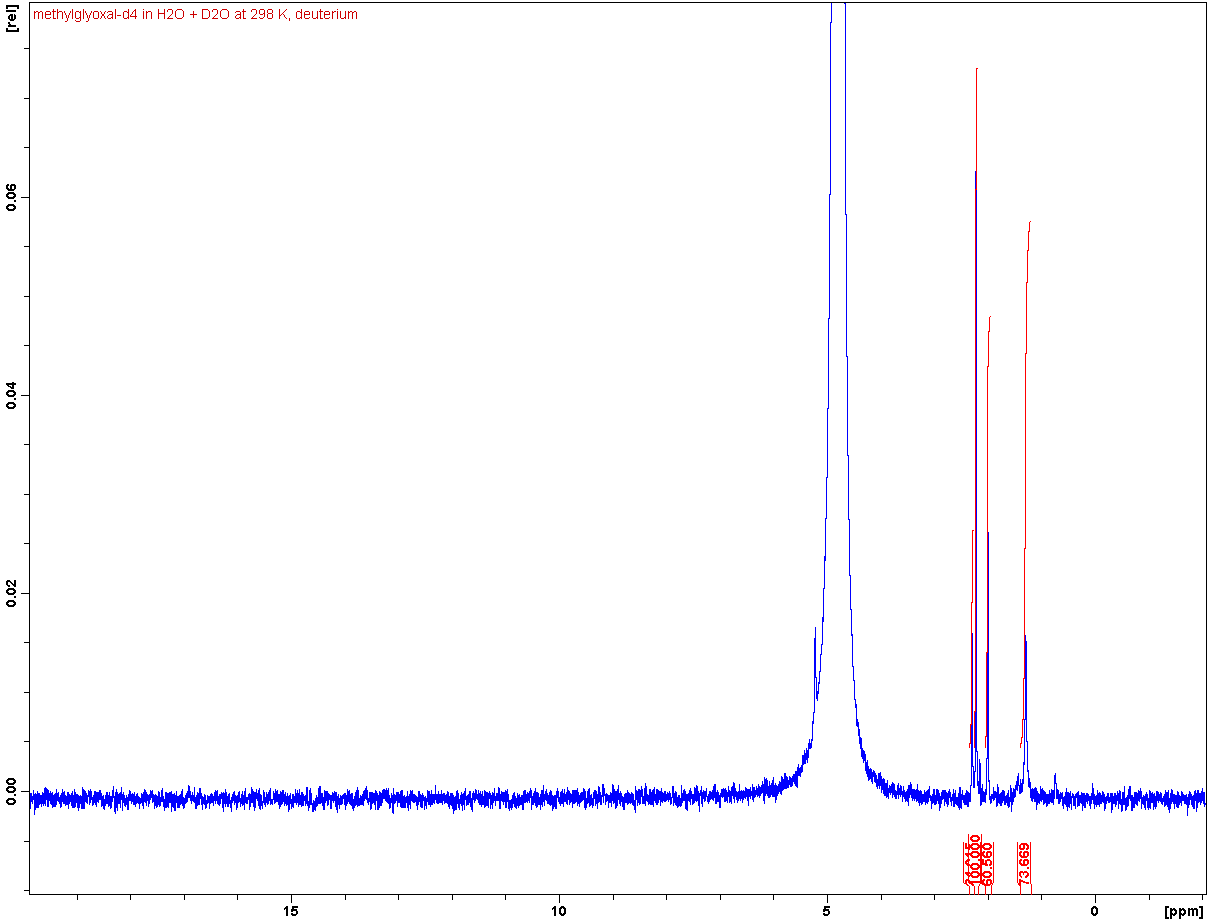


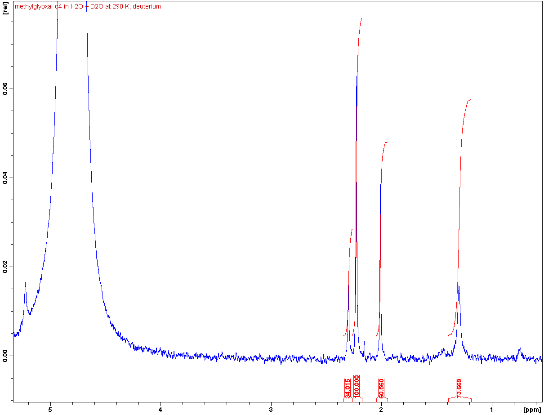
**A**

**
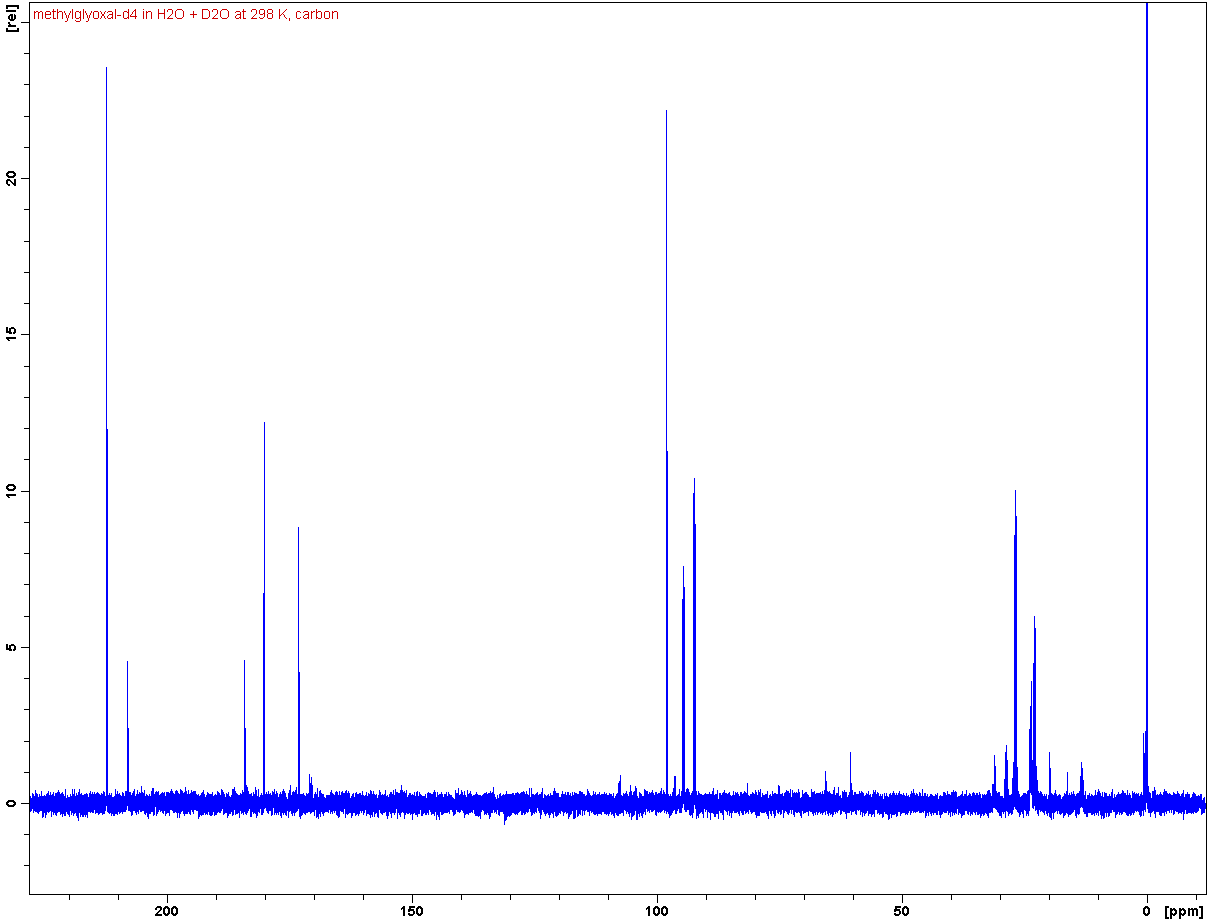
**

**B**

**Fig. S1** Representative (A) 2H-NMR and (B) 13C-NMR spectra of d4-methylglyoxal (d4-MG). D4-MG monohydrate: 2H NMR (298 K): 5.278 (s, H-1), 2.279 (s, 3H, H-3). 13C NMR (298 K): 212.20 (sp, JD,C = 1.0 Hz, C-2), 92.34 (t, JD,C = 25.1 Hz, C-1), 26.80 (sp, JD,C = 19.7 Hz, C-3); d4-MG dihydrate: 2H NMR (298 K): 1.349 (s, 3H, H-3), H-1 overlapped with the HDO signal. 13C NMR (298 K): 97.97 (C-2), 94.48 (t, JD,C = 24.9 Hz, C-1), 23.60 (sp, JD,C = 19.2 Hz, C-3).

**Fig. S2**

**
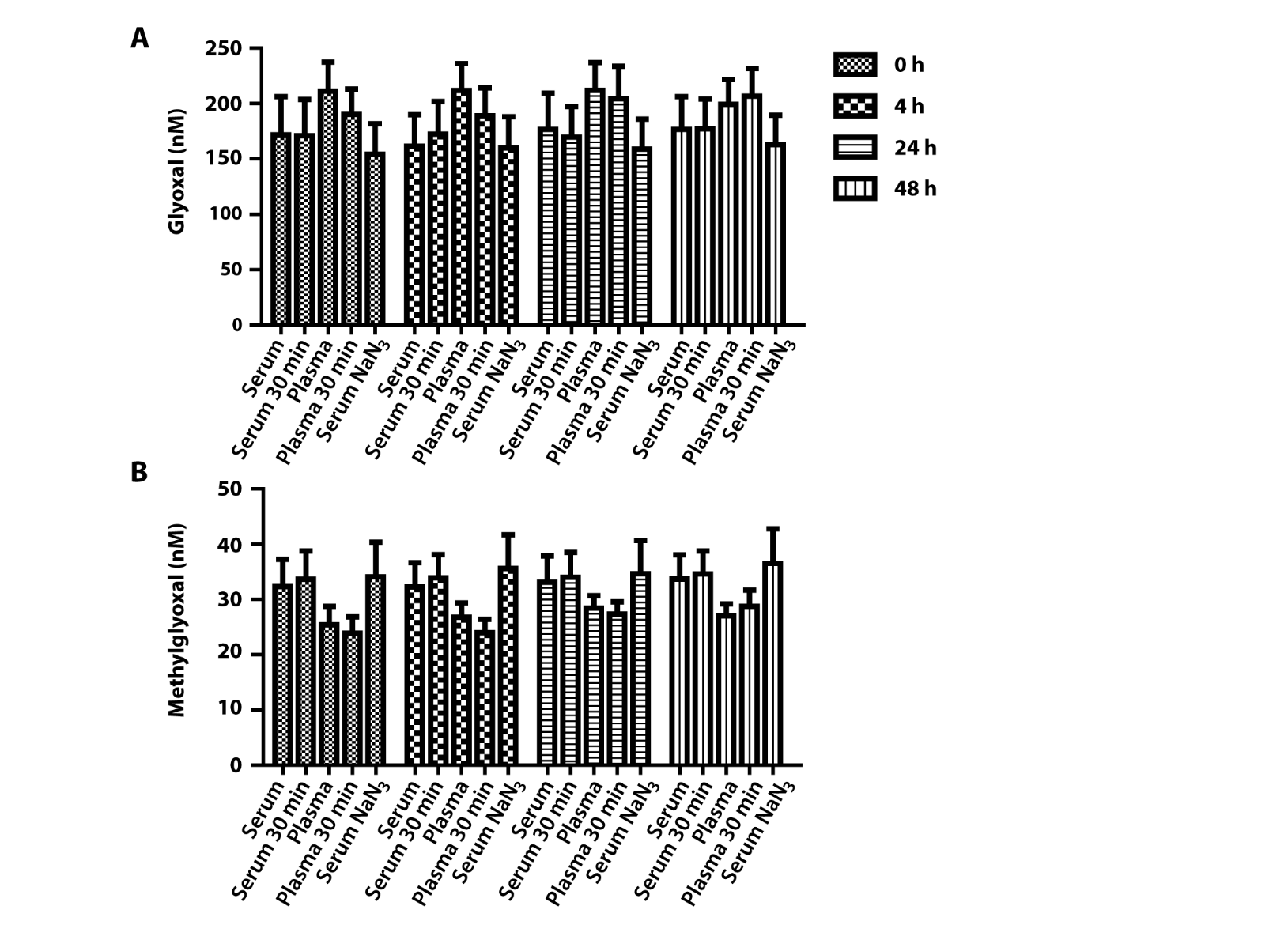
**

**Fig. S2** Stability of α-dicarbonyls. Investigation of the stability of glyoxal (A) and methylglyoxal (B) in blood samples with regard to sample preparation (serum/plasma), time and peroxidase inhibition with sodium azide.

**Pilot study to evaluate the stability of the α-dicarbonyls**

In order to investigate the stability of the highly reactive α-dicarbonyls, blood samples collected at the university hospital Schleswig-Holstein at different time points as well as differences between plasma and serum were analyzed. Therefore, after blood sampling, from each sample plasma (heparin) and serum (0 or 30 min at RT before centrifugation) were generated and samples were detected immediately or after 4 h, 24 h or 48 h at 4 °C. Furthermore, the effect of peroxidase inhibition with 0.3 % (w/v) sodium azide was analyzed as well. Subsequently, α-dicarbonyls were analyzed using mass spectrometry as described before.

**Fig. S3**


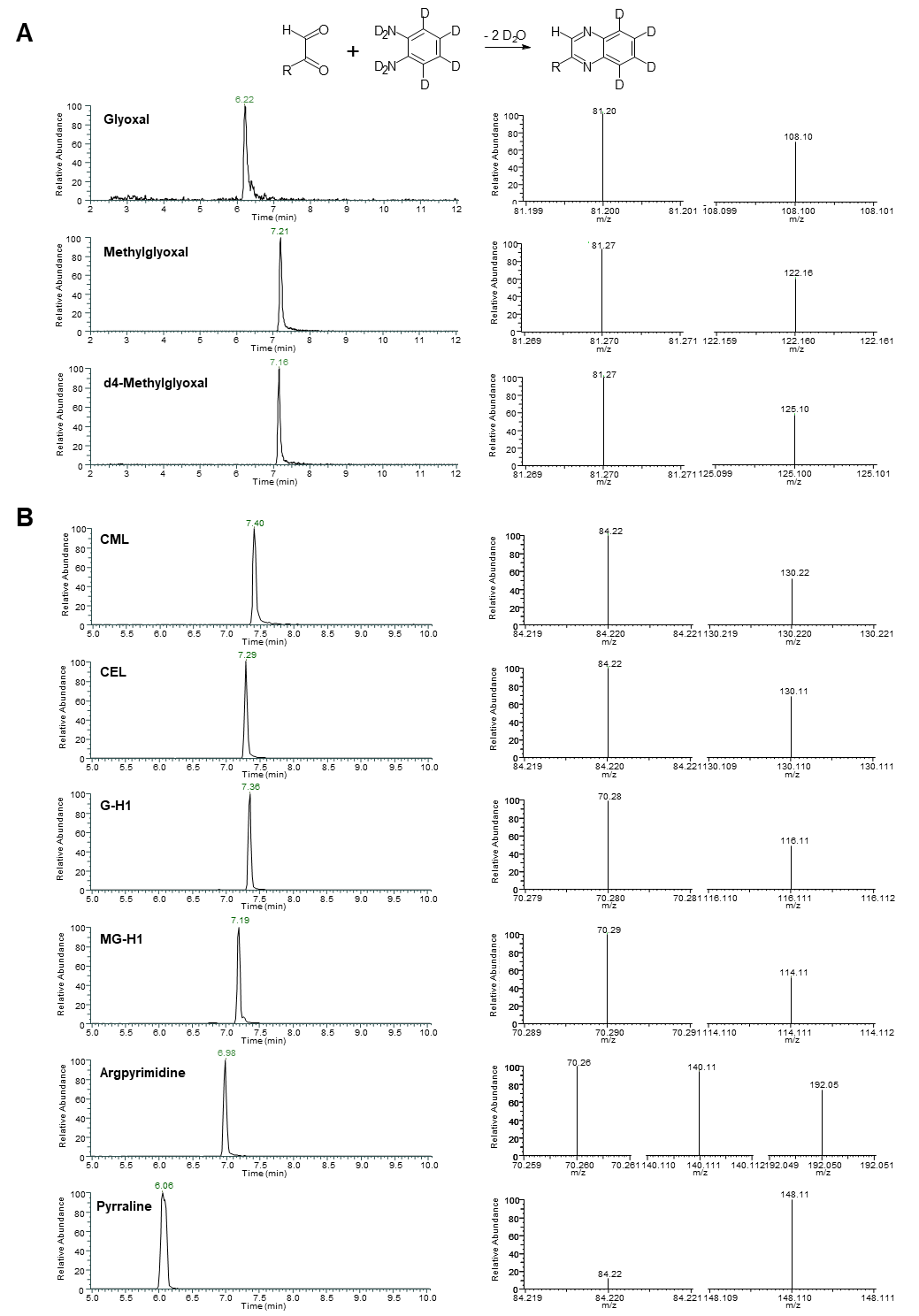


**Fig. S3** Representative LC-MS spectra and fragments of dicarbonyls (A) and glycated amino acids (B). The derivatisation reaction for the detection of dicarbonyls is presented in (A). R = H for glyoxal, R = CH_3_ for methylglyoxal.

**Fig. S4**

**Fig. S4** Glucose serum concentrations in cohort 1. Glucose serum concentrations in 80 non-diabetic and 22 diabetic patients were measured in venous blood samples on day 1. Patients without information regarding the diabetic status were excluded (n=2).

**Fig. S5**

**Fig. S5** Association of glycated amino acids with food intake of the patients in cohort 1. Correlation of pyrraline and MG-H1 with the time since the last meal of the patient before blood sampling, n=101 (A). Correlation of pyrraline with 4-hydroxyproline levels on day 3, n=86 (B). Spearman correlation; best-fit line with 95% confidence bands of slope. *, p<0.05; **, p<0.01; ***, p<0.001.

**Detection of 4-hydroxyproline**

Metabolic profiling was performed as described previously [1]. Briefly, 400 µl acetone/acetonitrile/methanol (1:1:1, v/v/v), containing 2.5 µM Metabolomics Amino Acid Mix Standard (Cambridge Isotope Laboratories, Andover, MA, United States), were added to 100 µl serum. After incubation and centrifugation, supernatants were dried under vacuum and reconstituted in 50 µl methanol/acetonitrile (1:1, v/v) for LC-MS/MS analysis. A Dionex Ultimate 3000 RS LC-system coupled to an Orbitrap mass spectrometer (QExactive, ThermoFisher Scientific, Germany) equipped with a heated-electrospray ionization (HESI-II) probe was used. Metabolites were separated on a SeQuant ZIC-HILIC column (150 × 2.1 mm, 5 μm) using water with 5 mM ammonium acetate as eluent A and acetonitrile/eluent A (95:5, v/v) as eluent B. The gradient elution was set as follows: isocratic step of 100% B for 3 min, 100% B to 60% B in 15 min, held for 5 min, returned to initial conditions in 5 min and held for 5 min. Flow rate was 0.5 ml/min. Data acquisition with data-dependent MS2 scans (top 10) was performed. 4-Hydroxyproline was identified by exact mass, retention time, fragmentation spectra and isotopic pattern based on an in-house library [1]. The area under the peak was normalized to the internal standard. Pooled samples at 5 concentrations were used as quality controls and for normalization. All solvents were of LC-MS grade quality and were purchased from Merck Millipore (Germany).

[1] Folberth, J. *et al*. MS2 and LC libraries for untargeted metabolomics: Enhancing method development and identification confidence. *J Chromatogr B,* 2020 May 15;1145:122105. doi: 10.1016/j.jchromb.2020.122105

**Fig. S6**

**
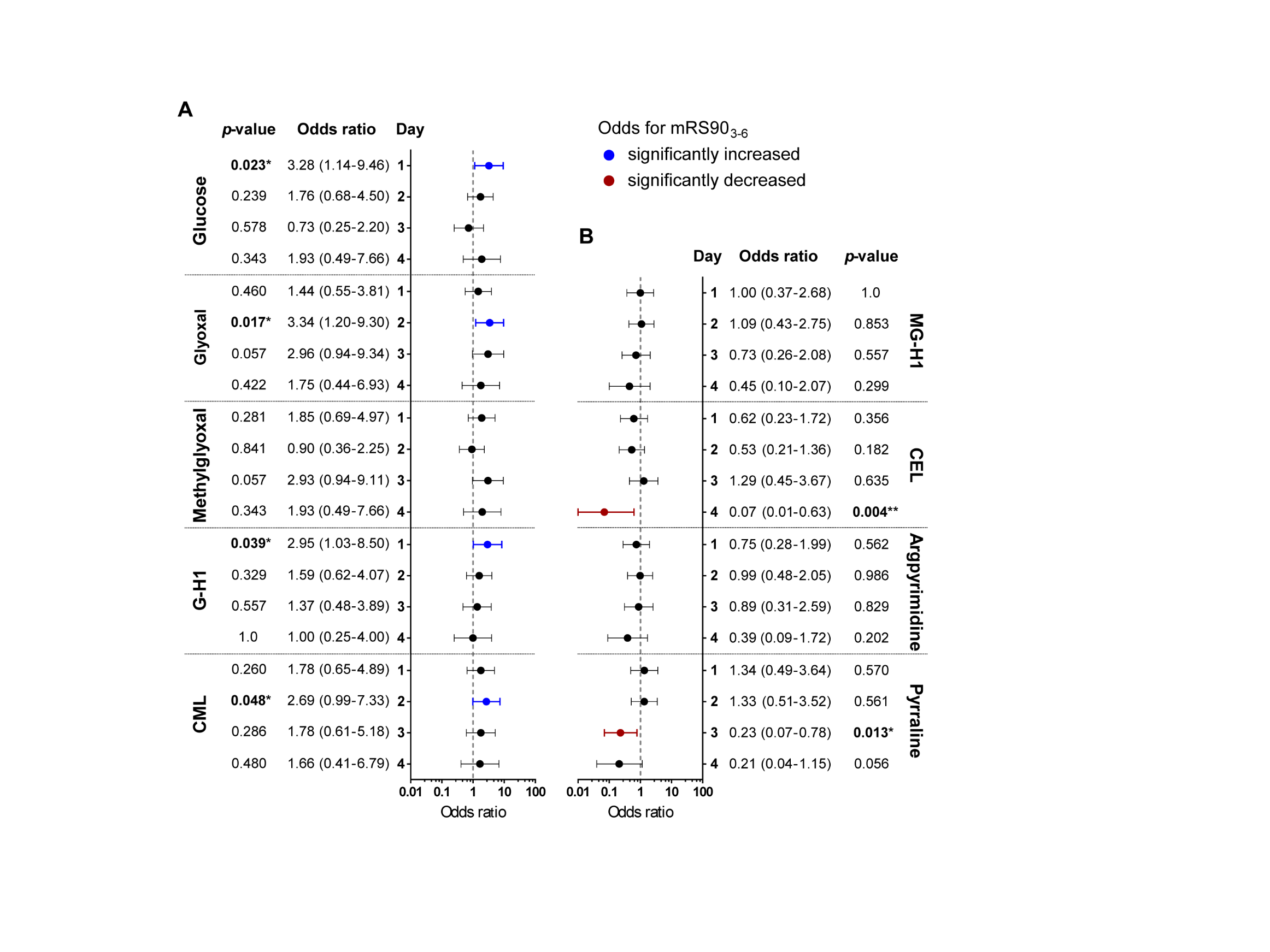
**

**Fig. S6** Forest plots of odds ratios for a bad outcome (mRS90 3-6) due to elevated serum levels of (A) glucose, α-dicarbonyls, and glyoxal-derived glycated amino acids or (B) glucose- or methylglyoxal-derived glycated amino acids on four consecutive days in cohort 1. Values are shown as OR (95 % CI) and statistical analysis was performed using the Chi squared-test; n=99 (day 1), n=108 (day 2), n=86 (day 3), n=51 (day 4).

**Fig. S7**


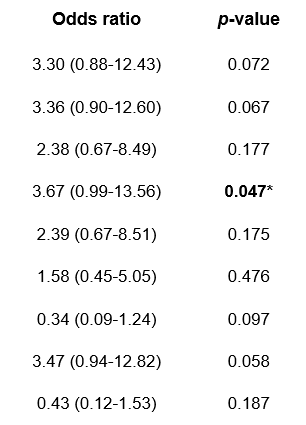


**Fig. S7** Forest plots of odds ratios for a bad outcome (mRS90 3-6) due to elevated serum levels of glucose, α-dicarbonyls, and glycated amino acids for cohort 2. Values are shown as OR (95 % CI) and statistical analysis was performed using the Chi squared-test; n=42. Blue marked data indicates significantly increased odds for mRS90_3-6_.

**Fig. S8**

**Fig. S8** Timeline of serum levels in patients from cohort 1 with a good or bad outcome. Concentrations of glycated amino acids were compared between good (mRS90_0-2_) and bad outcome groups (mRS90_3-6_). Mean ± SEM. *, p<0.05, **, p<0.01 (Mann Whitney U test).

**Fig. S9**

 **Fig. S9** Timeline of serum levels in non-diabetic patients from cohort 1 with a good or bad outcome. Concentrations of glucose, α-dicarbonyls and glycated amino acids were compared between good (mRS90_0-2_) and bad outcome groups (mRS90_3-6_). In contrast to the data presented in Fig. 3, patients with diagnosed diabetes mellitus were excluded. Mean ± SEM. *, p<0.05, **, p<0.01 (Mann Whitney U test).

**Fig. S10**

**Fig. S10** Comparison of patients with a good or bad outcome in cohort 2. Concentrations of glucose, α-dicarbonyls and glycated amino acids were compared between good (mRS90_0-2_) and bad outcome groups (mRS90_3-6_). Mean ± SEM. *, p<0.05, **, p<0.01 (Mann Whitney U test).

**Fig. S11**

**
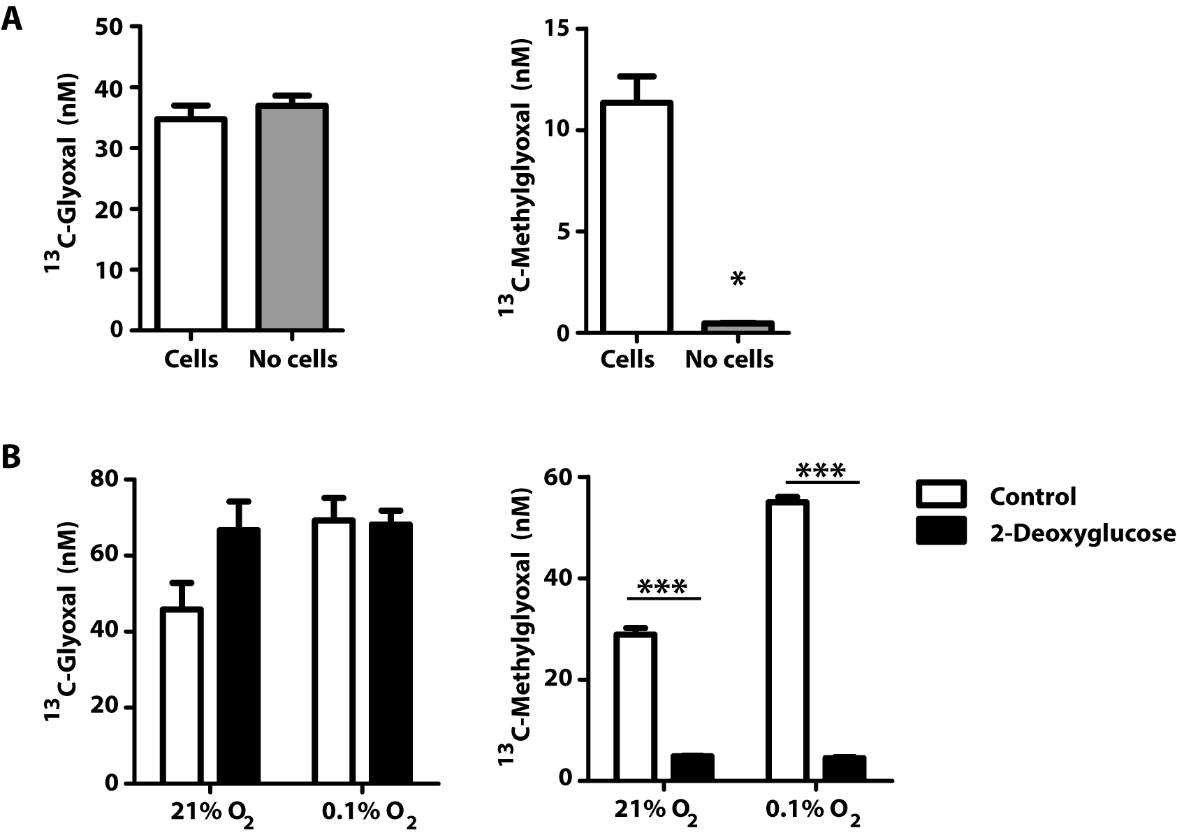
**

**Fig. S11** Association of 13C-labeled α-dicarbonyl production from ^13^C_6_-glucose with cellular metabolism. The dependence of the glyoxal and methylglyoxal formation on the presence of cells (A) or inhibition of glycolysis with 2-deoxyglucose (B). *, p<0.05, ***, p<0.01 (unpaired T test (A), 2-way ANOVA with Bonferroni post test (B)).

**Treatment of bEnd3 cells with 13C-glucose and 2-deoxyglucose**

The production of α-dicarbonyls from glucose was investigated using isotopically labeled ^13^C_6_-glucose and the inhibitor of glucose metabolism 2-deoxyglucose (2-DO). First, bEnd3 cells (ATCC) were cultivated for 6 h with ^13^C_6_-glucose, the medium collected and the concentration of ^13^C-labeled α-dicarbonyls was compared to the concentration in medium incubated without any cells. For the detection of ^13^C-labeled α-dicarbonyls the hybrid quadrupole-orbitrap mass spectrometer (Q‑Exactive, Thermo Scientific) has been used. The liquid chromatography, sample preparation and derivatization was done as described in the manuscript. The detection parameters have been adjusted to the increased mass according to the isotopic labeling (glyoxal M+3 m/z: 137.092; methylglyoxal: 152.111).

In order to investigate the connection of glucose metabolisation and dicarbonyl production, bEnd3 cells were treated with ^13^C_6_-glucose and 5 mM 2-DO or PBS. The α-dicarbonyls in the medium have been analyzed as described above.
